# Level of concern: COVID-19 threat and music preferences in college students

**DOI:** 10.3389/fpsyg.2026.1803349

**Published:** 2026-06-16

**Authors:** Terry F. Pettijohn, Molly H. Myers

**Affiliations:** Department of Psychology, Coastal Carolina University, Conway, SC, United States

**Keywords:** college students, COVID-19, environmental security hypothesis, listening to music, music

## Abstract

**Introduction:**

Music preferences can be influenced by a variety of factors, including feelings of environmental threat. The COVID-19 pandemic caused increased levels of stress and fear, which led to many behavioral changes in people’s everyday lives as they adjusted to the health crisis. For those more affected by the threat of the pandemic, music preferences may have changed, providing a unique opportunity to investigate musical preference influences.

**Methods:**

From August 2021 to May 2022, a sample of 161 college students completed online surveys regarding the level of threat they felt because of COVID-19 and what music they had been listening to, using music categories from the Short Test of Music Preferences (STOMP-R). We predicted participants who felt more threatened by COVID-19 would prefer to listen to reflective and complex music more (classical, jazz, folk, opera, international/foreign and blues music), consistent with previous research related to the Environmental Security Hypothesis.

**Results and discussion:**

Results followed these predictions and provided additional support for how the social environment can influence media preferences.

## Introduction

Coronavirus disease 2019 (COVID-19) is an illness caused by SARS-CoV-2 (severe acute respiratory syndrome coronavirus 2) (Mayo Clinic, 2025). COVID-19 was first identified in late 2019 and became a pandemic disease in 2020. The virus spread through the air in droplets of fluid passed from person to person in close contact. Some experienced no symptoms or mild illness while others, especially older individuals and adults with underlying medical conditions, required hospitalization and/or died. As of March 10, 2023, Johns Hopkins University reported 103,802,702 cases and 1,123,836 deaths from COVID-19 in the United States (Johns Hopkins University, 2026). The COVID-19 pandemic led to substantial changes in our everyday lives. During the pandemic, people had to isolate, keep their distance from people, cancel plans, and some lost jobs causing financial struggles. Many feared getting sick, dying, or losing a loved one to the virus. All of this added more stress and worry to our lives. Nearly half of all Americans reported experiencing some level of stress due to the pandemic and its effects (Keeter, 2020). The current study offers a preliminary exploration of the relationship between perceived threat from COVID-19 and musical preferences in college students.

Music plays a vital role in our everyday lives, often playing as we drive to work, workout in the gym, eat at restaurants, entertain friends at our homes, shop in stores, watch movies or television, or just relax by ourselves. It is estimated that people spend 3 h and 54 min on average per day listening to streaming music services, radio, podcasts, and satellite radio channels (Edison Research, 2025). In a recent international study of music listening habits, 71% of participants stated music was important to their mental health, 78% reported music helps them relax and cope with stress, and 87% reported they would like to listen to music to help them cope if they were in a hospital and in pain (International Federation of the Phonographic Industry, 2023). Effective positive and negative emotion regulation is important for maintaining mental and physical health and overall well-being (Kraiss et al., 2020; Tsujimoto et al., 2024). During times of crisis, people experience intense fears, anxiety, confusion, and a reduced sense of self-control (World Health Organization, 2025; Centers for Disease Control and Prevention, 2019). Music is a central component of our lives and can be used in important ways to help manage our emotions and health during threatening times and crises, such as the COVID-19 pandemic.

The Environmental Security Hypothesis (Nelson et al., 2007; Pettijohn and Tesser, 1999) contends that individuals prefer more meaningful, mature themes and items during tough social and economic conditions to help mitigate the threat and uncertainty. When times are more certain and less threatening, themes and items related to meaning and maturity should be less necessary; therefore, themes and items related to fun, celebration, and expression of carefree attitudes should be preferred. The COVID-19 pandemic was an international health threat that impacted normal living patterns. Fulkerson et al. (2021) found that there was a positive relationship between the COVID-19 pandemic and psychological distress, and students who felt a loss of social capital during the pandemic were especially distressed. During threatening times like these, people may consciously or unconsciously start listening to music that is slower, more meaningful, and comforting (Pettijohn and Sacco, 2009a; Pettijohn and Sacco, 2009b).

College students specifically experienced an abundance of added fear and uncertainty due to the pandemic. Many had to move back home from college, complete school online, and were separated from their college friends. During worrisome times, people help calm themselves by engaging in activities like exercising, meditating, writing, or listening to music (Vidas et al., 2021). Both international and domestic students from an Australian university reported listening to music as their most used form of coping (Vidas et al., 2021). College students often listen to music throughout their day, whether they are doing homework, walking to class, or just relaxing. When students feel threatened, they may listen to a specific type of music to cope with those feelings. Vidas et al. (2021) found that students tended to listen to music that was moderately negative in valence, meaning music that was angry or sad. This music was more meaningful compared to upbeat, fun music. Similar results were found by Pettijohn and Sacco (2009a) when the number one song from the *Billboard Top 100* for each year was compared to the socioeconomic climate of the year from 1955 to 2003. During threatening, or poor, socioeconomic years, people listened to music that was more comforting, romantic, meaningful, and slower (Pettijohn and Sacco, 2009a). Pettijohn et al. (2012) found slower pop songs and songs in less common keys are more reflective and serious, and these songs are more popular during social and economic bad times. In a thematic analysis of the lyrics of top *Billboard* songs from 1955–2003, during more difficult social and economic times, song lyrics featured more words, more personal pronouns, and more references to social processes, such as relationships (Pettijohn and Sacco, 2009b). Eastman and Pettijohn (2019) have also explored R&B/hip-hop music and found a similar pattern of results: when times are more challenging, longer, slower, and sadder songs are popular.

Putter et al. (2022) investigated popular music lyrics in the top charting weekly songs in the U.S. and the United Kingdom in the months before the COVID-19 pandemic and after it began. Lower satisfaction and human interest were found in the lyrics of popular songs during the first 6 months of the pandemic compared to songs from 2015–2019. Additionally, U.S. popular songs in 2020 had greater emotional content and researchers identified a positive relationship between economic misery and the number of negatively valanced words. A similar study was conducted (Yeung, 2020) comparing the top tracks on Spotify from six countries from January to July of 2020. Out of the six chosen countries, all experienced a strict lockdown due to the COVID-19 pandemic, except for Sweden. There was a significant correlation between fear of the pandemic and the strictness of the lockdown; the stricter the lockdown the more fear was reported. Along with these elevated levels of fear, people tended to listen to nostalgic music (Yeung, 2020). Nostalgic music may be more comforting than current music because it brings you back to perceived simpler, happier times from the past that may be especially soothing when experiencing a distressing health pandemic. Surviving a pandemic requires long-term psychological health strategies and resilience (Ding et al., 2025). Music can serve as an important tool to manage these emotions and mental health challenges (Golden et al., 2021; Gustavson et al., 2021).

Rentfrow and Gosling (2003) presented a useful structure for examining music preferences. They had participant’s rate music genres and identified four music preference dimensions. Reflective and complex (RC) is characterized by being slow in tempo, with mostly acoustic instruments, complex lyrics that express both positive and negative emotions, and low in energy. Intense and rebellious (IR) is described as having a fast tempo, electric instruments, moderately complex lyrics, and high in negative emotion and energy. Upbeat and conventional (UC) was reported to be simple and direct, high in positive emotion and energy, moderate in tempo, and using both acoustic and electric instruments. Energetic and rhythmic (ER) music is described by being moderate in tempo, use of eclectic instruments, somewhat complex lyrics, lack of emotion, and moderate in energy level. These four music preference dimensions were reviewed in relation to research on the Environmental Security Hypothesis. Past results from this area of research found slower, more meaningful, reflective, and serious music is preferred during conditions of social and economic threat. RC music is the only dimension of the four described as slow, which was a consistent finding in the Environmental Security Hypothesis music preference literature (Eastman and Pettijohn, 2019; Pettijohn et al., 2012; Pettijohn and Sacco, 2009a; Pettijohn and Sacco, 2009b). Additionally, we judged reflective and complex to be a good linguistic description of music that would be preferred during the threatening environmental conditions of a health pandemic. RC most closely aligned with the essential themes of slow, meaningful, serious music preferred during threating times, consistent with past Environmental Security Hypothesis music investigations. Therefore, we focused our predictions on RC music specifically.

Based on this past research, we predicted that college students who felt more threatened by the COVID-19 pandemic would prefer to listen to music that is more reflective and complex to help deal with the distressing environmental threat, consistent with the Environmental Security Hypothesis (Pettijohn and Sacco, 2009a; Pettijohn and Sacco, 2009b; Pettijohn and Tesser, 1999) and other research previously reviewed. While we did not make predictions about intense and rebellious, upbeat and conventional, or energetic and rhythmic music, we included these three other musical dimensions to explore their relation to COVID-19 threat. Musical choices are important ways college students can manage their feelings of threat and uncertainty.

## Methods

### Participants

Between August 2021 and April 2022 (Fall and Spring semesters), 163 college students were recruited from Introductory Psychology courses using SONA systems, an experimental participant management system, and offered 0.5 research credit points for participation (6 research credits required, or alternative activity). Two participants failed to complete most questions and were removed from the sample. An additional three participants skipped small portions of the surveys but were included for analyses where they completed the entire measure. The sample was primarily comprised of women (76%). With respect to race/ethnicity, the majority of the sample identified as white/Caucasian (77%), while 13% identified as Black/African American, 5.6% identified as Hispanic/Latino/Latina, 1.2% American Indian or Alaskan Native, 1.2% Native Hawaiian or other Pacific Islander, 0.6% Asian, and 1.2% other. The sample included students from a variety of majors. The majority of participants were first year students (60.9%), while 19.3% were sophomores, 9.3% juniors, 7.5% seniors, and 3.1% other. At the time of completing the online measures, 35.4% of participants had been diagnosed with COVID-19 previously, 88.2% had close friends of family members who had been diagnosed with COVID-19, and 19.3% had known a close friend or family member who had died of COVID-19. The majority of participants (67.7%) reported having received a COVID-19 vaccine, while 9.6% reported having a medical condition that restricted them from receiving a vaccine. The current study was reviewed and approved by the college institutional review board. American Psychological Association (2002) ethical guidelines were followed throughout the study.

### Materials and procedure

All participants completed the same set of online surveys, including the Social Psychological Measurements of COVID-19 (Conway III et al., 2020), the Short Test of Music Preferences-Revised (Rentfrow and Gosling, 2003; Rentfrow and Gosling, 2010; STOMP-R), and demographic questions, including age, sex, race/ethnicity, COVID-19 vaccine status, and other demographics.

The Social Psychological Measurements of COVID-19 (Conway III et al., 2020) is a scale designed to measure perceived coronavirus threat (PCT, 6 items), coronavirus impacts (CI, 9 items: financial, resource, and psychological), and coronavirus experiences (CE, 10 items: personal diagnoses/symptoms, proximity to others, and news). There are also federal, state, and city governmental responses to coronavirus questionnaires that were not utilized in the current study. All statements were rated using a 5-point Likert scale (*1 strongly disagree*, *5 strongly agree*).

The Short Test of Music Preferences-Revised (Rentfrow and Gosling, 2003; Rentfrow and Gosling, 2010; STOMP-R) separates 23 genres of music into four groups by creating labels that capture the main themes of the different genres of music. Participants rated each of the 23 genres on a 7-point Likert scale ranging from *Like Strongly* to *Dislike Strongly*. The four categories are reflective and complex (blues, jazz, classical, bluegrass, international/foreign, new age, opera, and folk music), intense and rebellious (rock, alternative, punk, and heavy metal music), upbeat and conventional (country, soundtrack, religious, gospel, soundtracks/theme songs, and pop music), and energetic and rhythmic (rap/hip-hop, funk, reggae, soul/R&B and dance/electronica music).

Participants agreed to online informed consent before answering any questions and read a debriefing statement when they were finished completing the online surveys.

## Results

Scores for perceived coronavirus threat (PCT) and music preferences for each music type (RC, IR, UC, and ER) were calculated. PCT was positively correlated with liking reflective and complex (RC) music, *r* (158) *= *0.21, *p = *0.009. The correlation between PCT and each of the three other music types (IR, ER, UC) were all near zero and not statistically significant. A statistically significant positive correlation was found between financial problems caused by COVID-19 and preferences for IR music, *r* (158) *= *0.17, *p = *0.04, and ER music, *r* (158) *= *0.17, *p = *0.03. Those who reported having close friends or family members struggle with COVID-19 reported an increased preference for UC music, *r* (158) *= *0.17, *p = *0.04. Those who consumed more COVID-19 news reported greater preferences for RC music, *r* (158) *= *0.21, *p = *0.007, as well as ER music, *r* (158) *= *0.16, *p =* 0.05. See Table 1 for results.

**Table 1:** Correlations between COVID-19 threat/subscales and music genre preferences.

Music genre categories				
Variable	R&C	I&R	U&C	E&R
PCT	0.207**	0.049	−0.071	0.050
CIF	0.151	0.165*	0.022	0.171*
CIR	0.017	0.026	0.004	0.048
CIP	0.074	0.066	0.021	−0.028
CEPD	−0.032	−0.128	0.149	0.075
CEPO	−0.033	0.024	0.165*	0.111
CEN	0.211**	0.118	−0.010	0.158*

*N* = 161; PCT, Perceived coronavirus threat; CIF, Coronavirus impacts financial; CIR, Coronavirus impacts resources; CIP, Coronavirus impacts psychological; CEPD, Coronavirus experiences personal diagnoses/symptoms; CEPO, Coronavirus experiences proximity to others; CEN, Coronavirus experiences news; R&C, Reflective and complex; I&R, Intense and rebellious; U&C, Upbeat and conventional; E&R, Energetic and rhythmic.

^*^p < 0.05. ^**^p < 0.01.

Investigating music genres individually, PCT was positively correlated with preferences for classical [*r* (157) = 0.22, *p =* 0.006], opera [*r* (156) = 0.26, *p =* 0.001], international/foreign [*r* (158) = 0.18, *p* = 0.024], and jazz music [*r* (156) = 0.20, *p* = 0.011]. PCT was negatively correlated with preferences for country music, *r* (158) = −0.20, *p* = 0.012. These were the only significant correlations between PCT and individual music genres. When asked to select their favorite music genre during the pandemic, the most popular genres selected were Rap/Hip Hop (27.5%), Pop (16.9%), Country (12.5%), Alternative (11.3%), and Soul/R&B (6.9%).

Within the COVID-19 threat measure subscales for coronavirus impacts and coronavirus experiences (Conway III et al., 2020), a strong positive correlation was found between those impacted financially and those whose resources were impacted by COVID-19, *r* (159) *= *0.352, *p < *0.001. Greater perceived impacts of close family or friends by COVID-19 was positively correlated with greater experienced psychological impacts of COVID-19, *r* (159) *= *0.22, *p = *0.005, and greater personal diagnoses and symptoms of COVID-19, *r* (159) *= *0.43, *p < *0.001. Those who consumed more COVID-19 related news had a strong positive correlation with psychological impacts of COVID-19, *r* (159) *= *0.32, *p < *0.001, greater COVID-19 financial impacts, *r* (159) *= *0.30, *p* < 0.001, and greater COVID-19 impacts on resources, *r* (159) *= *0.16, *p* = 0.05.

In other exploratory analyses, men (*n* = 38) reported a lower level of PCT than women, *t* (158) = 2.61, *p* < 0.01. There were no significant differences between men and women when exploring music preferences. Additionally, those who received the coronavirus vaccine also reported a higher level of PCT, *t* (159) = 3.74, *p* < 0.001. Analyses were also completed separately for fall and spring semester. Participant demographics were very similar between semesters, and analyses all followed the same pattern of outcomes.

## Discussion

This study provides preliminary correlational evidence of a relationship between college students’ perceived threat towards COVID-19 and music preference. It was hypothesized that those who reported a higher level of COVID-19 threat would prefer more reflective and complex music from the Short Test of Music Preferences-Revised (Rentfrow and Gosling, 2003; Rentfrow and Gosling, 2010; STOMP-R). After analyzing the data that was collected, the correlation between PCT and preference of RC music was statistically significant, supporting our predictions and the Environmental Security Hypothesis (Nelson et al., 2007; Pettijohn and Sacco, 2009a; Pettijohn and Sacco, 2009b; Pettijohn and Tesser, 1999). Increased PCT was positively related to preferences for music that is more reflective and complex. Although the correlation between PCT and RC was statistically significant, we only accounted for 4.4% of the variance. While this is a small amount of variance accounted for, we understand many factors determine music preferences. PCT was not correlated with the other music genre categories: IR, UC, or ER. When looking at the individual genres of music, there was a positive correlation found between PCT and classical, opera, international/foreign, and jazz music. These genres all fall under the reflective and complex category of music because they tend to be slow in tempo, use acoustic instruments, have complex lyrics, and are low in energy. Additionally, there was a negative correlation found between country music preference and PCT. Those who reported a preference for country music also reported less threat from COVID-19. Eastman and Pettijohn (2015) studied popular country music hits across social and economic times and found country music was different from pop music trends. The most popular country songs of the year were more upbeat, lyrically positive, and used more happy-sounding major chords during more difficult times. If country music consumed during the COVID-19 pandemic followed these same patterns, it would make sense that those who were less concerned with the virus would prefer country music with its more positive tone and message.

We recognize limitations of the current study. A cross-sectional convenience sample of college students from the Southeastern United States was used. This limited sample prevents us from generalizing to the population of college students and the general public of all music listeners. As stated previously, the sample was diverse with respect to where they were from in the U.S., their academic majors, and year in school, but future studies could further explore regional differences, sex differences, and adult populations outside a college environment. In addition, we understand the limitations of online survey research. Students were not monitored as they provided responses, but we are not aware of any false information being provided or response bias in the sample. We also explained that our study took place during 2021–2022, which was after the initial COVID-19 pandemic started. While this was not the beginning of the pandemic, it was still a period of high uncertainty and concern for many students. Longitudinal data from the start to the end of the COVID-19 pandemic would have been ideal to determine true shifts in musical preferences. We also relied on musical categories from Rentfrow and Goslings’s (2003, 2010) STOMP-R, which are well-validated but other scales to measure musical preferences could be used.

It should be noted that Rap/Hip Hop (27.5%) was the most popular music genre in our study, but this genre preference was not related to PCT. It would appear that COVID-19 threat was not related to preference for Rap/Hip Hop, although this genre was classified as EC and not part of our original predictions. While only about a quarter of the students selected Rap/Hip Hop as their favorite, these results also show there was not one dominant favorite genre. In addition, a favorite genre may be problematic to provide and interpret, as students often report liking a diverse collection of musical selections and they listen to various genres at different times to manage their moods and emotions. Asking how much students like a variety of genres, as we did in the current study, provides additional information than just ascertaining a single favorite genre of music. We do not suggest that students only listened to RC music during the pandemic, but that they listened to RC music *more* if they felt threatened by COVID-19. While the categories provide a good overall pattern of musical preferences with COVID-19 threat, individual songs within other genres may have included meaningful themes, slower music, and complex ideas. While other research has explored some of these individual trends during the pandemic (Putter et al., 2022; Yeung, 2020), these musical selections relating to COVID-19 threat were not specifically investigated.

Future extensions of this research may also use experimental methods to measure music preferences in research labs after making health threats salient. This could be done by manipulating negative news surrounding COVID-19 or another health risk, then measuring participant level of perceived threat toward the health risk and musical preferences. Pettijohn and colleagues (Pettijohn and Sacco, 2009a; Pettijohn et al., 2014), also explored the features of the artists who were making the music. When times were more difficult, artists with more mature facial features were more popular. In a future study, it would be interesting to explore popular artist age, sex, and facial features during the COVID-19 pandemic to see if performer characteristics have an influence in artist popularity within a health crisis.

Despite the potential limitations, these findings align with previous research connecting environmental threat to preferences for slower, more meaningful music (Eastman and Pettijohn, 2019; Pettijohn and Sacco, 2009a; Pettijohn and Sacco, 2009b). COVID-19 has been tough for most students and those who reported being most threatened by COVID-19 reported preferring music that was more meaningful, reflective, and complex in the current study. In April of 2020, during the COVID-19 pandemic, the musical duo Twenty One Pilots released the song *Level of Concern* (Joseph, 2020). The song explored some of the chaos, fear, and anxiety experienced during the pandemic, along with a hopeful message of making it through the challenging time by seeking comfort and assurance from those we love. This was an important message during the coronavirus pandemic. *Level of Concern* provides a good example of the type of music that was created during the pandemic, about the experience of the COVID-19 pandemic, which resonated with listeners as they navigated a new reality. These general study results also agree with the Vidas et al. (2021) study results where students experiencing stress due to COVID-19 preferred music that was negative in valence, meaning its lyrics were complex and negative in emotion. Music negative in emotion falls under the category of reflective and complex music. The findings are also consistent with previous research that shows differences in the song selections before and during the pandemic (Putter et al., 2022) and the Spotify lists focusing on nostalgic music (Yeung, 2020). Music can help reduce negative emotions, stress, and anxiety (Groarke et al., 2020; Jiang et al., 2016; Labbé et al., 2007), which is especially important during a viral pandemic.

The results of the current study offer preliminary support for the current hypothesis, and additional general support for the Environmental Security Hypothesis (Nelson et al., 2007; Pettijohn and Sacco, 2009a; Pettijohn and Sacco, 2009b; Pettijohn and Tesser, 1999). The Environmental Security Hypothesis may be applied to help individuals manage their own emotions during periods of stress and uncertainty, as well as in counseling settings. The therapist could try having reflective and complex music playing softly in the background before or during sessions to ease anxiety and distress. This may help the patient feel more relaxed or comfortable while they are in the professional’s office. The counselor could also try recommending to their patients to listen to RC music when they are in stressful situations outside of their session. Another use of the results in this study could be in the field of marketing and advertisements. Advertisers can use this information to select songs to match the mood of the consumer to make products more desirable. These results may also be valuable if we were to experience a future health pandemic collectively, or individual threatening situations. Knowing musical preferences can change with social and economic conditions, artists may create new music that matches the mood of the nation to increase their appeal. People can also pick and choose the type of media that may help them navigate their current individual situation. The interplay between feelings of environmental threat and music preferences is an important topic that deserves additional investigation in the future.

## Data Availability

The raw data supporting the conclusions of this article will be made available by the authors, without undue reservation.

## Author’s note

Portions of this research were presented at the Annual Association for Psychological Science Convention in Washington, DC, May 2023.
